# Human kidney pericytes produce renin

**DOI:** 10.1016/j.kint.2016.07.035

**Published:** 2016-12

**Authors:** Ania Stefanska, Christopher Kenyon, Helen C. Christian, Charlotte Buckley, Isaac Shaw, John J. Mullins, Bruno Péault

**Affiliations:** 1 University/BHF Centre for Cardiovascular Science, University of Edinburgh, Edinburgh, UK; 2 MRC Centre for Regenerative Medicine, University of Edinburgh, Edinburgh, Scotland, UK; 3 MRC Centre for Inflammation Research, University of Edinburgh, Edinburgh, UK; 4 Department of Physiology, Anatomy and Genetics, University of Oxford, Oxford, UK; 5 Orthopaedic Hospital Research Center, University of California, Los Angeles, California, USA

**Keywords:** CD146, mesenchymal stem cell, nephrogenesis, NG2, pericyte, renin, tissue RAS

## Abstract

Pericytes, perivascular cells embedded in the microvascular wall, are crucial for vascular homeostasis. These cells also play diverse roles in tissue development and regeneration as multi-lineage progenitors, immunomodulatory cells and as sources of trophic factors. Here, we establish that pericytes are renin producing cells in the human kidney. Renin was localized by immunohistochemistry in CD146 and NG2 expressing pericytes, surrounding juxtaglomerular and afferent arterioles. Similar to pericytes from other organs, CD146^+^CD34^–^CD45^–^CD56^–^ renal fetal pericytes, sorted by flow cytometry, exhibited tri-lineage mesodermal differentiation potential *in vitro*. Additionally, renin expression was triggered in cultured kidney pericytes by cyclic AMP as confirmed by immuno-electron microscopy, and secretion of enzymatically functional renin, capable of generating angiotensin I. Pericytes derived from second trimester human placenta also expressed renin in an inducible fashion although the renin activity was much lower than in renal pericytes. Thus, our results confirm and extend the recently discovered developmental plasticity of microvascular pericytes, and may open new perspectives to the therapeutic regulation of the renin-angiotensin system.

Pericytes (also known as mural cells) wrap around microvessels and play a crucial role in vascular development, maintenance, and remodeling.^1^, ^2^, ^3^ Reciprocal communication between pericytes and endothelial cells exists because they directly interact through a shared basement membrane and long cytoplasmic processes that ensheath the vessel wall.^4^ Specialized types of renal pericytes contribute mechanically to vascular tone and blood pressure regulation,^5^, ^6^ and can modulate immune responses.^7^, ^8^ Crisan *et al*.^9^ prospectively identified human vascular pericytes from multiple human organs based on the expression of CD146 (Mel-CAM), nerve/glial antigen 2 (NG2), and platelet-derived growth factor receptor-β (PDGFR-β), and demonstrated that pericytes are one of the origins of mesenchymal stem cells (MSCs). Pericytes show regenerative potential in injured skeletal muscle,^9^, ^10^ ischemic heart,^11^, ^12^ bone,^13^, ^14^ adipose tissue,^15^ and dental pulp^16^; therefore, they are regarded as a local reservoir of regenerative cells.^17^, ^18^ In tissue fibrosis, pericytes have a pathological role in which they proliferate and differentiate into collagen-I–producing myofibroblasts.^19^, ^20^ In aging, kidney pericyte loss leads to capillary dilation and vascular damage.^21^

Cells of renin lineage (CoRL), which originate from renin-positive precursors, include smooth muscle, mesangial, and some epithelial cells.^22^ Recently, a role for CoRL in disease has become evident. CoRL have been shown to regenerate parietal epithelial cells and/or podocytes in a podocyte depletion model,^23^ erythropoietin-producing cells in chronic hypoxia,^24^ and mesangial cells after injury.^25^

CoRL and pericytes share gene expression and transcription factor regulatory circuits. Gene expression analysis of CoRL isolated from renin-reporter mice shows enrichment in the pericyte marker regulator of G-protein signaling 5.^26^ Furthermore, the RBP-J transcriptional modulator is crucial for both renin expression and pericyte development.^27^, ^28^ Deletion of RBP-J causes a reduction in renin mRNA in the kidney, with decreases in circulating renin levels and blood pressure, and an inability to recruit renin-expressing cells when homeostasis is threatened.^27^ Pericytes, which are Foxd1^+^ cell derivatives,^29^ are severely affected by targeted disruption of RBP-J in the Foxd1 lineage.^28^ FOXD1^RBPJ−/−^ mice die prematurely and show a decreased number of renal arteries and arterioles, and an absence of glomerular cells associated with extensive glomerulosclerosis postnatally. Recent studies of the lineage relationships revealed that all mural cells, including renin-expressing cells, are derived from Foxd1^+^ stromal cells.^30^

We hypothesized that the versatile nature of renin-expressing cells is related to their perivascular identity. Therefore, we investigated the developmental affiliation between pericytes and renin-expressing and/or -producing cells in the human kidney. For the first time, human kidney pericytes were typified, purified, cultured, and functionally characterized as MSCs. Principally, we showed that fetal renal pericytes natively express renin with functional enzyme activity.

## Results

### Identification of pericytes in the human fetal kidney

**Figure 1:**
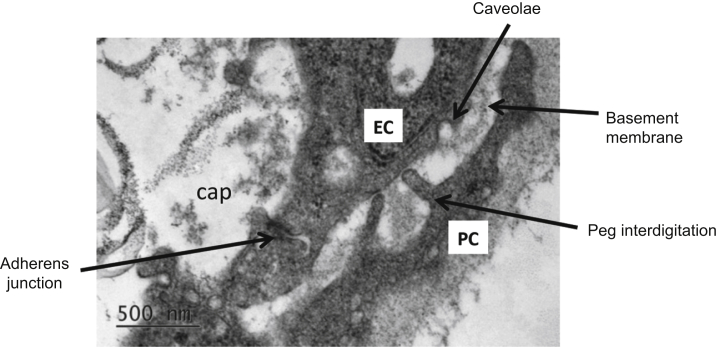
**Electron micrograph illustrating peg-socket assembly between a pericyte and an endothelial cell in a 10-week-old human fetal kidney.** The pericyte is partly submerged into the capillary basement membrane and extends the “peg” processes to contact the endothelial cell (EC). A shared basement membrane is present between the pericyte and the EC. cap, capillary lumen; PC, pericyte. Bar = 500 nm.

**Figure 2:**
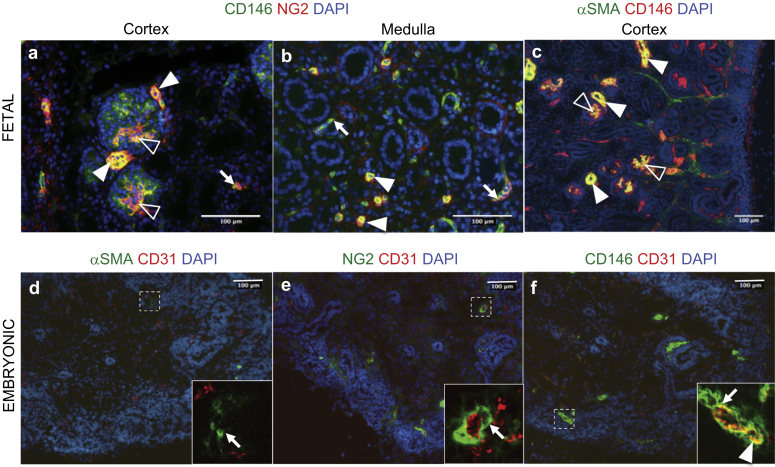
**Identification of pericytes in human embryonic and fetal kidneys.** (**a**) Fetal pericytes were labeled with antibodies against CD146 (green) and NG2 (red) in a 15-week-old kidney cortex. CD146^+^NG2^+^ pericytes were found in peritubular capillaries (arrow), mesangial cells (open arrowheads), and afferent arterioles (arrowheads). (**b**) In the kidney medulla, pericytes encircled peritubular capillaries (arrows) and the vasa recta (arrowheads). (**c**) Double staining for CD146 (red) and smooth muscle α-actin (αSMA) (green) shows that in the cortex, pericytes coexpress αSMA in the mesangium (open arrowheads) and afferent arterioles (arrowheads). In embryonic pericytes, sections of 7-week-old kidneys were stained for pericyte markers: CD146 (green), nerve/glial antigen 2 (NG2) (green), and αSMA (green), with the endothelial cell marker CD31 (red) (on consecutive sections). (**d**) αSMA immunoreactivity was sparse; only single vascular smooth muscle cells were found (inset, arrow). (**e**) NG2^+^ cells surrounded endothelial cells (inset, arrow). (**f**) CD146 staining was perivascular (inset, arrow) and in endothelial cells (inset, arrowhead). DAPI, 4′,6-diamidino-2-phenylindole.

Pericyte identification on tissue sections requires (i) confirmation of vascular anatomy, (ii) localization of ≥2 pericyte markers, and (iii) counterstaining of an endothelial cell marker.^31^ First, we identified pericytes in the human developing kidney by transmission electron microscopy (TEM). An ultrathin section of the fetal kidney shows “peg and socket” anchoring between pericytes and adjacent endothelial cells (Figure 1). Pericytes are partially embedded into the capillary basement membrane; therefore, they can be distinguished from fibroblasts that do not establish specific contacts with endothelial cells.^32^ Secondly, we used CD146 and NG2 coexpression to label pericytes. In the human fetal kidney cortex, the pericyte markers CD146 and NG2 were localized to the mesangium, afferent arterioles, and interstitial capillaries (Figure 2a). In the medulla, pericytes were found in the vasa recta and peritubular capillaries (Figure 2b).

It has long been known that certain pericyte populations, such as those in the vasa recta, are associated with α-smooth muscle actin (αSMA) expression and contractility.^5^, ^33^ Pericytes and vascular smooth muscle cells (αSMA^+^ cells) are related by their mesenchymal origin and are not readily distinguished from each other.^4^ Transitional pericytes and/or vascular smooth muscle cells have been documented in arterioles and venules.^2^, ^34^ To examine αSMA expression in relation to pericyte markers, cells were costained for αSMA and CD146. In the cortex, CD146^+^αSMA^+^ cells were detected in afferent arterioles and mesangium (Figure 2c), but they were not detected in the medulla (not shown).

Next, we queried whether αSMA, NG2, and CD146 expression overlap at earlier stages of kidney development. Cells were costained with CD31 to visualize endothelium. Few αSMA expressing cells were observed in the 7-week-old kidney (Figure 2d). NG2 and CD146 expressions were more pronounced, both marking the developing vasculature (Figure 2e and f). Taken together, these results indicate that CD146 and NG2 expression precedes that of αSMA in human kidney development.

### Renin-expressing cells in the human fetal and adult kidney are pericytes

**Figure 3:**
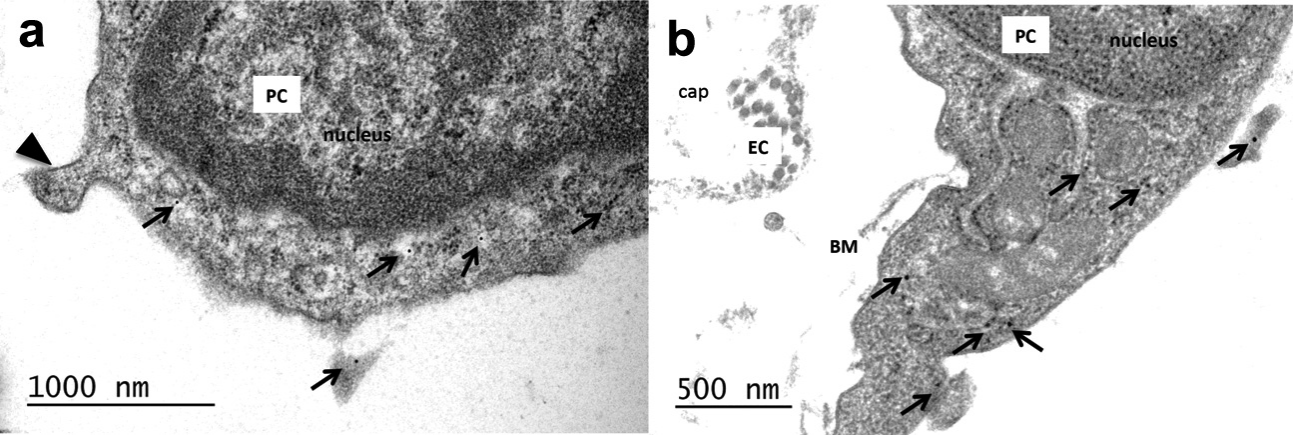
**Immunogold labeling of renin in human kidney pericytes (14-week-old kidney).** (**a–b**) Multiple renin granules were visualized with 10-nm immunogold particles (arrows) in the pericyte cytoplasm. Arrowhead indicates a pericyte peg. BM, basement membrane; cap, capillary lumen; EC, endothelial cell; PC, pericyte. Bar = 1000 nm in (**a**) and 500 nm in (**b**).

**Figure 4:**
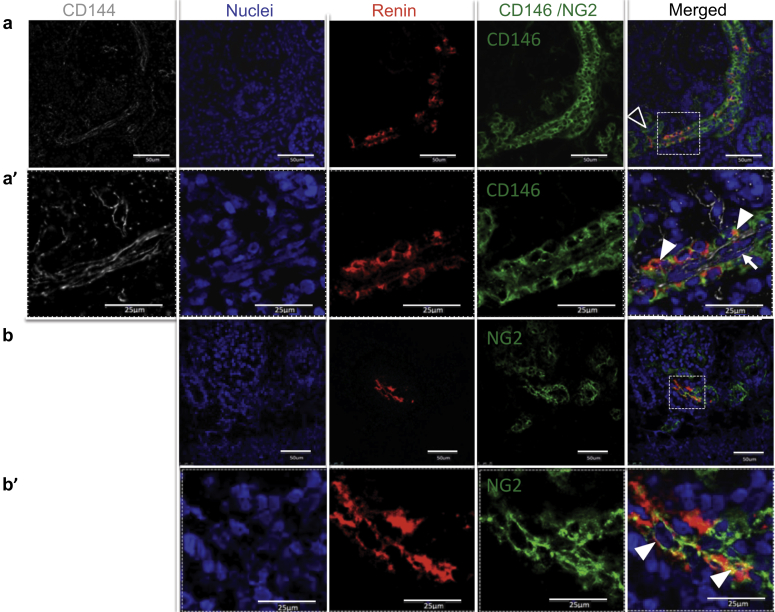
**Renin is colocalized with pericyte markers in the human fetal kidney.** Triple staining of fetal kidney demonstrates that (**a**) renin immunoreactivity (red) coincides with pericyte marker CD146 (green) expression in afferent arterioles. CD146 staining is also present in the mesangium (open arrowhead). Characteristically, renin staining had a striped pattern. The inset (**a′**) shows an afferent arteriole at high magnification. Renin and CD146 stainings overlap on the abluminal side of the vessel (arrowheads), whereas immunoreactivity for CD144 (grey), an endothelial cell marker, is visible inside blood vessels (arrow). (**b**) Nerve/glial antigen 2 (NG2) (green) staining is found in the mesangium and afferent arteriole. Renin (red) is present in the JG area. Inset (**b′**) shows renin^+^ cells coexpressing NG2 (arrowheads) at higher magnification. JG, juxtaglomerular cell.

**Figure 5:**
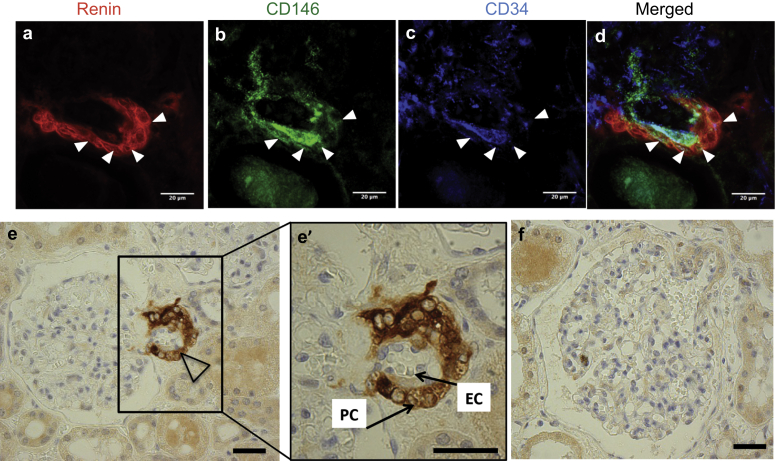
**Renin-expressing cells in the human adult kidney are pericytes.** Triple staining of human adult kidney sections shows that (**a**) renin-expressing cells (red) are colocalized with (**b**) CD146 pericyte marker (green) expression (arrowheads) in afferent arterioles, whereas (**c**) renin-expressing cells do not express the CD34 endothelial cell marker. (**d**) The merged image illustrates the overlap between renin and CD146 expressions, indicated by arrowheads. (**e**) Transverse section of the JG area in the adult kidney demonstrates renin immunoreactivity in an afferent arteriole (open arrowhead). Inset (**e′**) shows JG vascular structure at higher magnification. On the abluminal side of the vessel, pericytes (PC) (arrow) show characteristic ring-like distribution in the vascular wall, which coincides with renin expression. Endothelial cells (EC) are localized to the luminal side of the vessel (arrow). (**f**) No primary antibody control is included that showing negligible staining. Bars = 1 μm on (**e**), (**e′**), and (**f**). JG, juxtaglomerular cell.

First, TEM renin immunolabeling was used to determine renin expression *in vivo*. Immunogold labeling revealed numerous gold particles in the pericyte cytoplasm of fetal kidneys (Figure 3a and b). Second, confocal microscopy images for renin, CD146, and NG2 in the fetal kidney were obtained. CD146 staining was found in the mesangium and afferent arterioles, and renin was localized to the juxtaglomerular (JG) area and afferent arterioles, which presented a characteristic striped pattern of expression (Figure 4a). Coexpression of renin and CD146 was apparent on the abluminal side of the vessel (Figure 4a′), and none of the renin^+^ cells expressed the endothelial marker CD144 (Figure 4a′). NG2 staining was present in the mesangium and afferent arterioles; renin was colocalized in afferent arterioles (Figure 4b and b′). Third, renin immunoreactivity was investigated in adult kidney sections. Similar to the fetal kidney, renin in afferent arterioles coincided with the pericyte marker CD146, but not with endothelial CD34 (Figure 5a–d). In addition, a close look at the morphology of renin-expressing arterioles revealed that renin was restricted to the abluminal side of the vessel, but it was absent from the endothelial luminal surface (Figure 5e and f). In conclusion, we found that renin-expressing cells are pericytes *in vivo*.

### Purification of pericytes from human fetal kidney

**Figure 6:**
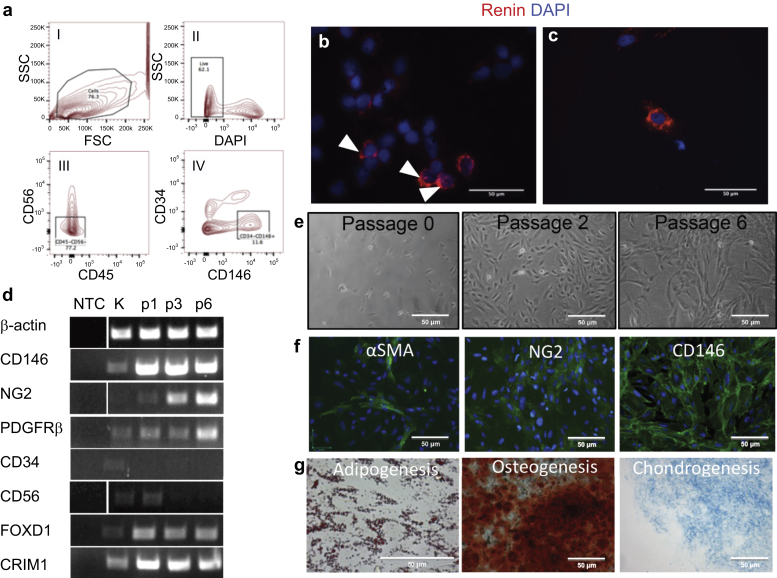
**Isolation and *in vitro* characterization of pericytes from a human fetal kidney.** (**a**) FACS of renal pericytes purified from a 16-week human fetal kidney. Dot plots show backgating strategy to obtain CD146^+^CD34^−^CD56^−^CD45^−^ pericytes. Red-color dots show gated populations and the percentages of the parent gates. The sorted pericyte population contained no CD45^+^, CD56^+,^ or CD34^+^ cells. (**b**) Cytospins of freshly sorted pericytes were immunostained for renin (red, arrowheads). (**c**) Granular, intracellular expression of renin (red) can be observed for up to 48 hours in occasional cells of renal pericyte primary cultures. Positively stained cells are native JG cells, which retain renin expression for a short term *in vitro*. (**d**) Representative results of gene expression profiles of renal pericyte primary cultures (n ≥ 3). Pericytes showing expression of the pericyte markers CD146, nerve/glial antigen 2 (NG2), and platelet-derived growth factor receptor-β (PDGFRβ), were negative for hematopoietic CD45 and metanephric mesenchyme CD56 expressions. In addition, pericyte cultures were enriched in Foxd1 (stromal marker) and CRIM1 (pericyte/parietal cell/podocyte marker). (**e**) Morphology of cultured CD146^+^CD34^−^CD56^−^CD45^−^ pericytes. Bright field images of renal pericyte primary cultures are shown after plating at passages (p) 0, 2, and 6. Cells displayed a spindle-shaped morphology at p0 and p2 but eventually acquired fibroblastic-like appearance at p6. (**f**) Cultured kidney pericytes at p2 show some α-smooth muscle actin (αSMA) (green) positive staining, whereas most of the cells are positive for NG2 (green) and all cultured pericytes are CD146^+^ (green). (**g**) Kidney pericyte primary cultures underwent mesodermal lineage induction. After 14 days of differentiation, primary cultures were confirmed to differentiate along adipogenic (Oil red O staining), chondrogenic (Alcian blue staining), and osteogenic lineages (Alizarin red staining). Control cells were incubated with growth medium (not shown). Original magnifications are shown on the images. DAPI, 4′,6-diamidino-2-phenylindole; FACS, fluorescence-activated cell sorter; FSC, forward scatter; JG, juxtaglomerular cell; K, kidney (positive control); NTC, no template control; SSC, side scatter.

Primary cultures of fetal kidney cells were established to investigate the renin induction potential of kidney pericytes. A method for pericyte purification by flow cytometry from multiple human tissues has been described previously.^35^, ^36^ The gating strategy is shown in Figure 6a. CD146^+^CD34^−^CD56^−^CD45^−^ pericytes were sorted at an average percentage from total live cells of 4.15 ± 2.61% (n = 22).

Cytospun kidney pericytes were analyzed for renin immunoreactivity. Antirenin staining confirmed that freshly isolated pericytes included renin-expressing cells (Figure 6b), and that renin immunoreactivity was retained in culture; 0.003 ± 0.002% of cells were renin-positive after 48 hours (Figure 6c).

### Phenotyping human renal pericytes

Semiquantitative real-time polymerase chain reaction (RT-PCR) analysis showed that renal pericyte primary cultures express high, stable levels of CD146 and gradually increasing levels of NG2 and PDGFR-β across passages 1 to 6. Cultured pericytes were negative for CD34. Depletion of endothelial cells in sorted pericytes was based on (i) a CD34 negative selection on the fluorescence-activated cell sorter, (ii) additional negative selection of endothelial cells using CD144 in most of the sorts performed for this study (not shown), and (iii) verification by flow cytometry that renal pericyte cultures did not contain endothelial cells (CD31^+^) (not shown). CD56 was detected at passage 1 of sorted pericytes, but was not detected at later passages. We also confirmed that the FOXD1 stromal cell marker^29^ was present in cultured pericytes as well as CRIM1, which plays a role in maintaining renal microvascular integrity^37^ (Figure 6d).

During the initial phase of cell culture, pericytes displayed elongated, spindle-shaped morphology (Figure 6e, passage 0). However, after a few weeks in high glucose medium, cells displayed a more rounded shape (Figure 6e, passage 2). At passages 5 to 6, renal pericytes appeared homogenously elongated, with extended cell processes (Figure 6e, passage 6).

Immunocytochemistry performed at passage 2 showed that cultured kidney pericytes contained low levels of αSMA with a high frequency of NG2^+^ cells and ubiquitous CD146 immunoreactivity (Figure 6f).

MSCs are defined by their ability to differentiate into several mesodermal cell lineages. After 2 weeks, pericyte primary cultures with lineage-specific differentiating media exhibited features of adipogenic osteogenic or chondrogenic cell lineages (Figure 6g).

### Cultured human fetal renal pericytes produce renin

**Figure S1:**
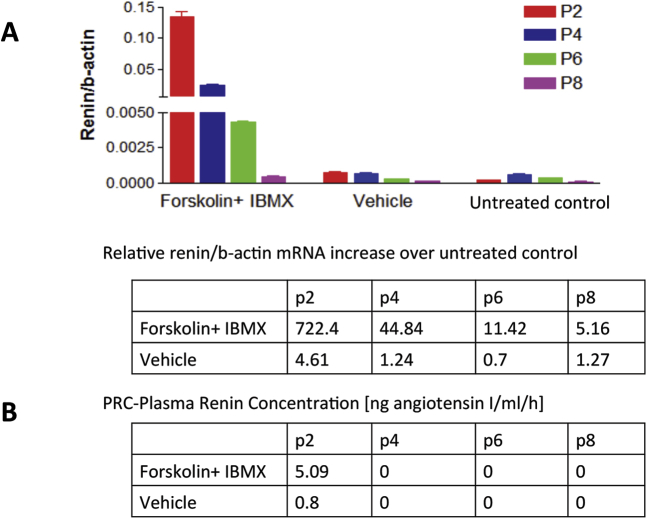
Diminished renin induction response in the primary cultures of human kidney pericytes over time. Renal pericytes isolated from the fetal human kidney upregulate renin expression when treated with cyclic adenosine monophosphate inducers. Cells underwent renin induction at different passages (p2, p4, p6, and p8). (**A**) Quantitative polymerase chain reaction (PCR) demonstrated a dramatic reduction in the ability to re-induce renin over increasing number of culture passages. (**B**) Renin activity assay showed an enzymatic activity present at passage 2 (p2) only. Data are shown as means ± SEM (n = 1). Error bars are calculated from technical replicates. IBMX, isobutyl-1-methylxanthine.

**Figure 7:**
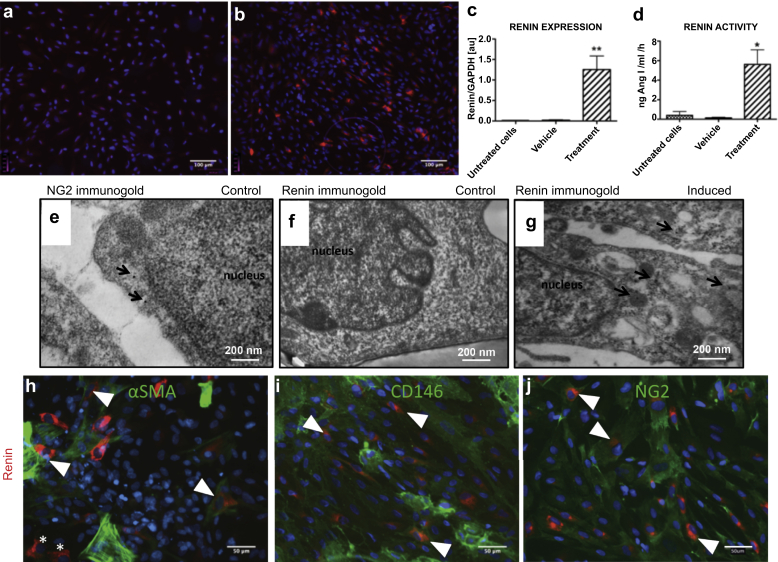
**CD146^+^NG2^+^ αSMA^+/−^ renal pericytes express and secrete enzymatically active renin *in vitro*.** Renal pericyte primary cultures at passage 2 were stained for renin (red). (**a**) Control cells (vehicle: medium + vehicle; untreated cells: medium) show no renin immunoreactivity. Forskolin and isobutyl-1-methylxanthine (cyclic adenosine monophosphate induction) treatment in pericytes (**b**) caused robust renin staining, (**c**) increased renin mRNA expression, and (**d**) renin activity. One-way analysis of variance followed by Bonferroni’s *post hoc* comparisons were used to test statistical significance. Data are shown as mean ± SEM (n = 3, *P < 0.5; n = 5, **P < 0.01). Electron micrographs of human pericytes showed immuno-gold labeling for nerve/glial antigen 2 (NG2) (arrows) (**e**) but (**f**) no renin immuno-gold labeling. (**g**) Forskolin-treated pericyte showing immuno-gold labeling for human renin (arrows indicate the immuno-gold label). Renin-induced kidney pericyte primary cultures stained positively for pericyte markers: CD146, NG2, α-smooth muscle actin (αSMA) (all green), and renin (red). (**h**) Renin expression was not always correlated with αSMA expression (65.68 ± 7.4% overlap). Double positive cells for renin and αSMA are marked with arrowheads and renin^+^αSMA^−^ cells are indicated by an asterisk. Renin is co-expressed unequivocally (100% overlap) with (**i**) CD146 and (**j**) NG2. Double positive cells for renin and pericyte markers are marked with arrowheads. GAPDH, glyceraldehyde-3-phosphate dehydrogenase.

To demonstrate that renal pericytes have the potential to produce and secrete renin, primary cell cultures were assayed for renin expression, immunoreactivity, and enzymatic activity after treatment with cyclic adenosine monophosphate (cAMP) inducers: forskolin and isobutyl-1-methylxanthine (IBMX).^38^, ^39^ Control cells (vehicle: medium + vehicle; untreated cells: medium) demonstrated no renin immunoreactivity (Figure 7a), whereas 4.60 ± 2.50% of induced cells expressed renin (Figure 7b). Induced renal pericyte primary cultures showed substantial upregulation of renin expression after 24 hours (Figure 7c). Renin mRNA was virtually absent in untreated (0.01 ± 0.00) and vehicle-treated cells (0.02 ± 0.01) compared with cells treated with cAMP inducers (n = 5; 1.25 ± 0.33; *P* < 0.01). A renin activity assay confirmed that pericytes produce metabolically active renin (Figure 7d). In medium from control cells, renin activity was virtually undetectable (0.40 ± 0.40 and 0.02 ± 0.12 ng angiotensin I/ml/h in untreated and vehicle cells, respectively) compared with activity in medium from induced cells (n = 3; 5.62 ± 1.49 ng angiotensin I/ml/h; *P* < 0.05). In agreement with previous studies,^26^, ^40^ it was noted that early passaged cells displayed greater potential for renin induction. Induction of renin expression and activity showed a dramatic decline beyond passage 2 (Supplementary Figure S1).

TEM confirmed the presence of renin granules in induced kidney pericytes. Human pericyte ultrastructure was well preserved, and immunogold labeling for the pericyte marker NG2 was localized to the cytoplasm (Figure 7e). Immunogold labeling of renin was visible in pericytes treated with cAMP inducers (Figure 7g) but not in noninduced control cells (Figure 7f). The cytoplasm of induced pericytes appeared more vacuolated (Figure 7g) compared with controls (Figure 7e and f).

### Renin-expressing cells are CD146^+^NG2^+^αSMA^+/−^ cells

To verify that renin-producing cells were pericytes, primary cultures of cells were stained for renin and CD146, NG2, and αSMA; 65.68 ± 7.40% of cells were renin^+^/αSMA^+^ (Figure 7h), and all renin^+^ cells were costained for CD146 (Figure 7i) and NG2 (Figure 7j). Therefore, renin-expressing cells in these experiments were defined as CD146^+^NG2^+^αSMA^+/−^. We concluded that NG2 is a better marker for the renin-producing cell population, because *in vivo* NG2 is mainly associated with arterioles (where renin is expressed) and capillaries.^41^

### Nonrenal pericytes also express and produce renin

**Figure S2:**
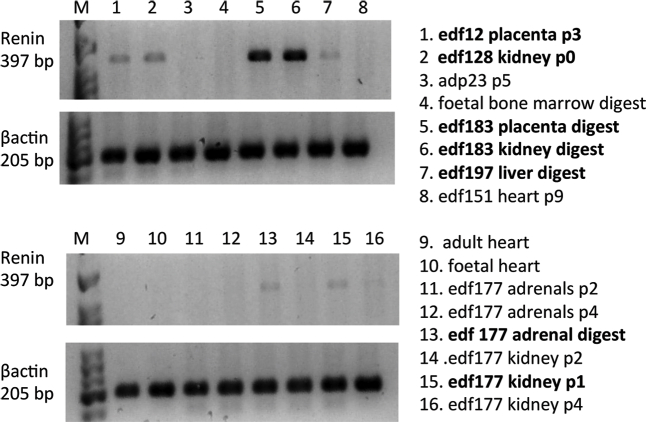
Renin gene expressions in renal and nonrenal tissue digests, and primary pericyte cultures. Among tested samples, the highest renin expression was present in the kidney and placenta digests, followed by kidney and placenta-derived cultured pericytes. Detectable renin expression was also found in the adrenal digest. β-actin was used as a housekeeping gene for real-time polymerase chain reaction. M, marker; p, passage.

Components of the renin angiotensin system (RAS) have been found in many human tissues and are commonly referred to as local RASs.^42^ We hypothesized that renin induction potential is an intrinsic feature of pericytes of renal and nonrenal origins. Renin gene expression was therefore compared in tissues and primary cultures of pericytes derived from second trimester tissues: fetal kidney, liver, adrenal glands, and placenta. High renin expression was found in fetal kidney and placenta tissue digests, lower levels of expression were present in cultured renal and placental pericytes, and the least amount was detected in fetal liver and adrenal gland digests (Supplementary Figure S2).

**Figure 8:**
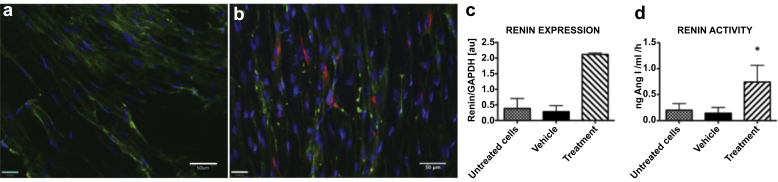
**Inducible renin expression in primary placental pericytes.** Second trimester placental pericyte primary cultures were stained for renin (red) and pericyte marker nerve/glial antigen 2 (green). (**a**) Control cells (vehicle: medium + vehicle; untreated cells: medium) did not stain for renin. In contrast, (**b**) forskolin and isobutyl-1-methylxanthine–treated cells show renin immunoreactivity. Cyclic adenosine monophosphate AMP induction results in (**c**) renin mRNA upregulation; however, (**d**) renin activity is modest. Data are shown as mean *±* SEM (n = 2 for renin expression, n = 3 for renin activity, ∗*P* < 0.5). GAPDH, glyceraldehyde-3-phosphate. dehydrogenase.

Placental pericytes show renin immunoreactivity *in vitro* after incubation with cAMP inducers (Figure 8a and b). No renin positivity (0%) was observed in control cells, whereas 4.64 ± 2.02% of induced cells were positive. Primary placental pericytes had increased renin mRNA levels after 24-hour treatment with cAMP inducers (Figure 8c). Renin mRNA levels were low in untreated (0.38 ± 0.32), and vehicle-treated (0.28 ± 0.28) cells, but were significant after induction (2.11 ± 0.05; n = 2). Renin activity was measured in culture medium from primary cells after renin induction and was increased (0.74 ± 0.32; n = 3) compared with untreated (0.20 ± 0.13) and vehicle-treated cells (0.14 ± 0.1 ng angiotensin I/ml/h). However, renin gene expression did not correlate with renin activity (Figure 8d).

## Discussion

This study provided definitive evidence that renin-producing cells are—at least some of them—pericytes. Previously, a lineage relationship between renin-expressing cells and pericytes was proposed based on microarray studies,^26^ and recently, it was shown that renin-expressing cells and pericytes are derived from a common Foxd1^+^ progenitor.^30^ We used a human fetal kidney to demonstrate that renin-expressing cells are pericytes, as defined by anatomic distribution and surface marker expression. We determined that primary cultures of isolated kidney pericytes contained renin-expressing cells that, when induced, responded by increased renin mRNA expression, protein production, and secretion of active renin. Pericytes isolated from nonrenal tissues were also shown to express renin in an inducible manner.

Our data confirmed and extended previous reports on the affiliation between renin-expressing cells and pericytes by providing *in vitro* evidence of the existence of a distinguished subset of microvascular pericytes that natively express renin. Previously, fate-tracking studies showed that during development, renin-expressing cells give rise to mesangial, arteriolar, and interstitial cells that can resume renin expression when stressed.^22^, ^43^ Plasticity of the renin cells is a great advantage in adapting to environment changes and maintaining homeostasis.

Developmentally, renin-producing cells are derived from the metanephric mesenchyme.^44^ Sequeira Lopez *et al.*^44^ demonstrated, by single-cell nested PCR, that early renin-expressing cells express the mesenchymal marker Ets1 but not αSMA, which is acquired later in development. This agrees with our results, which showed αSMA expression as a late event compared with the onset of renin and pericyte marker expression. A small subset of pericytes in all human tissues analyzed do not express αSMA^17^ and have been interpreted as being the most primitive ones in terms of regenerative potential, although no marker has yet been identified to purify these cells. We confirmed that pericytes that led to renin-producing cells in the embryonic and/or fetal kidney are mostly (or exclusively) αSMA-negative. It will be important to determine whether renin-producing cells can be also recruited from αSMA-negative pericytes from the adult kidney or other organs.

Studying JG cells, which natively produce renin, remains a difficult task for several reasons: (i) there is no JG specific marker except for renin; (ii) they are rare (0.01%–0.001% of the total kidney cell mass); and (iii) renin production is switched off after 48 to 72 hours in culture.^26^, ^40^ *In vitro* studies support the mesenchymal origin of renin-producing cells. 3T3 pre-adipocytes^39^ and murine MSCs differentiate into renin-producing αSMA^+^ cells^45^ when treated with cAMP inducers (forskolin and IBMX). CD44^+^ MSC-like cells respond to dietary sodium depletion and captopril administration by activation and differentiation into renin-producing cells; however, only a small percentage of recruited renin lineage cells coexpress CD44.^46^

The so-called extra-renal tissue RAS has been described in multiple organs, including the adrenal glands, heart, brain, vascular wall, reproductive tract, skin, digestive organs, sensory organs, and lymphatic tissue.^42^, ^47^, ^48^ Our findings suggest a role of pericytes in promoting extra-renal RAS. Tissue RASs display differences compared with the circulating RAS in terms of functions and regulation. Most of tissue renin is released under an inactive form; however, it is believed that small amounts of angiotensin II can be generated in an autocrine fashion within tissues.^49^, ^50^ All key components of the RAS are expressed in the human placenta.^51^, ^52^ Immunohistochemical staining showed renin distribution in the placental vasculature and syncytiotrophoblast.^53^ However, most (92%–96%) of released renin is inactive.^54^, ^55^ Numerous studies have demonstrated that the highest levels of renin and/or prorenin are detected during the first trimester and reduce to low levels at term.^54^, ^56^, ^57^, ^58^ High levels of renin, prorenin receptor, and the AT1 receptor in early gestation have been correlated with expression of vascular endothelial growth factor, which suggests a role for RAS in placental angiogenesis and trophoblast proliferation and invasion.^58^

A canonical feature of cultured pericytes from multiple tissue origins is tri-lineage mesodermal differentiation ability.^9^ We also observed this for the first time in renal pericytes, which implies that the kidney can also be a source of MSCs. It is tempting to suggest that renin secretion ability represents another facet of the broad developmental potential of ubiquitous pericytes. In this respect, it will be important to assess renin secretion by pericytes isolated from tissues that do not contain a RAS. Conversely, the potential to express renin may be specific to pericytes from the kidney and other organs hosting a RAS. Little is known regarding tissue-related restrictions in the regenerative and/or regulatory potential of pericytes. Importantly, the developmental origin of pericytes is varied; most are derived from mesoderm, but those in the cephalic region stem from the neural crest,^31^ which could indicate as yet undiscovered significant functional differences. Localized pericyte surface markers have been also described, which might indicate different tissue-specific functions.^59^ So far, the best-documented example of an anatomic restriction is the cardiac pericyte. Cardiac pericytes, unlike those from other tissues,^9^, ^60^, ^61^ cannot differentiate into skeletal muscle, but can, to some extent, give rise to cardiomyocytes.^12^ Overall, our results support the concept that pericytes serve as a reservoir of tissue-specific progenitors.^62^

The present study is the first to describe the isolation and characterization of human renal pericytes. We demonstrate that isolated kidney pericytes, like those from other organs, have MSC properties *in vitro*. Kidney-derived pericytes also exhibit distinctive renin expression and secretion. However, nonrenal pericytes can also be induced to produce renin, which supports the unifying concept of the existence of perivascular tissue RASs. This novel hypothesis opens the field for further research into the physiological and pathophysiological regulation of renin-inducible pericytes and their potential therapeutic role.

## Materials and Methods

### Human tissue collection

Human kidneys (6–18 weeks of development) and second trimester placenta were obtained after elective abortions, with informed consent from the donor and in compliance with the rules established by the South East Scotland Research Ethics Committee. Developmental age was estimated from the crown to rump distance measurements and medical history. Eight weeks of development is the limit between embryonic and fetal life in humans.

Normal human adult kidney biopsies were obtained from nephrectomy.

### Tissue processing for cell sorting

Tissues were minced and incubated in Dulbecco’s modified Eagle medium (DMEM; Invitrogen, Paisley, UK)/20% fetal bovine serum (FBS; Invitrogen)/1% penicillin, streptomycin (PS; Invitrogen) with 0.5 mg/ml of each collagenase (IA, II, and IV; Sigma-Aldrich, Dorset, UK) at 37 °C in a shaking water bath. After a 70-minute incubation, dispersed cells were passed through 100- and 70-μm filters (Fisher Scientific, Loughborough, UK) and centrifuged (10 minutes at room temperature). Cells were resuspended in red blood cell lysis buffer (9:1 ratio of stock 1 [8.3-g ammonium chloride/L water]; stock 2 [20.59-g Tris base/LH20]) at 37 °C for 5 minutes, then washed, resuspended in phosphate-buffered saline (PBS; Invitrogen)/2% FBS/1% PS, and filtered through a 40-μm cell strainer.

Cell suspensions (approximately 10^8^/ml) were incubated on ice for 20 minutes in the dark with fluorescence-conjugated antibodies (diluted 1:100): CD146-Alexa Fluor 647 (Abd Serotec, Kidlington, UK), and CD34-PE, CD45-APC-Cy7, and CD56-PE-Cy7 (BD Biosciences, Oxford, UK). The initial step for each flow analysis was to create a standard side scatter/forward scatter dot plot to eliminate cell aggregates, debris, and dead cells. Dead cells were excluded from the analysis using 4′,6-diamidino-2-phenylindole (DAPI) (Invitrogen). Kidney pericytes were sorted as a CD146^+^CD34^−^CD45^−^CD56^−^ population. Placental pericytes were obtained using the same protocol. Cell sorting was performed using a FACSAria flow cytometer (BD Biosciences).

### Primary cell culture of kidney pericytes

Isolated pericytes were seeded in endothelial cell growth medium-2 (EGM-2, Cambrex, Bio Science, Wokingham, UK) on 0.2% gelatin-coated culture dishes (Sigma-Aldrich) at a cell density of 2 × 10^4^ cells/cm^2^ and cultured at 37 °C, with 5% carbon dioxide. After reaching confluence, cells were passaged and maintained in high glucose DMEM/20% FBS/1% PS.

Multilineage differentiation assays to promote osteogenic, adipogenic, and chondrogenic phenotypes were performed as described previously.^9^, ^12^

### Real-time polymerase chain reaction and quantitative real-time polymerase chain reaction

RNA was extracted using the phenol-chloroform method,^63^ and cDNA was generated with a SuperScript III Reverse Transcriptase system kit (Invitrogen). RT-PCR was performed with Taq DNA polymerase (Bioline) and carried out using primers listed in Supplementary Table S1.

Quantitative PCR was performed with Universal Probe Library System Technology (Roche Applied Science, Welwyn Garden City, UK). The following primers were used: glyceraldehyde-3-phosphate dehydrogenase: forward 5′-AGCCACATCGCTCAGACAC-3′; glyceraldehyde-3-phosphate dehydrogenase reverse 5′-GCCCAATACGACCAAATCC-3′; renin forward 5′-TACCTTTGGTCTCCCGACAG-3′; and renin reverse 5′- TTGAGGGCATTCTCTTGAGG-3′ (Eurofins MWG Operon, Ebersberg, Germany).

### Immunohistochemistry and immunocytochemistry

Human developing kidneys were snap-frozen in optimal cutting temperature freezing medium. Tissue sections were fixed in acetone/methanol (1:1) for 10 minutes, then incubated at 4 °C overnight with the following antihuman primary antibodies: anti-CD146 (BD Pharmingen 1:100); anti-CD146-FITC (AbD Serotec 1:100); anti-NG2 (BD Pharmingen 1:300); anti-αSMA-FITC (Sigma 1:200); anti-renin (R&D Systems 1:100; R&D Systems, Abingdon, UK); anti-CD144 (Thermo Scientific 1:200); and anti-CD31 (Abcam 1:300). This was followed by 1-hour incubation with secondary antibodies conjugated with Alexa Fluor 488, 555, or 647 (Invitrogen 1:1000). To preserve the sections and visualize nuclei, DAPI-mounting medium (DAPI-Fluoromount-G; SouthernBiotech, Cambridge, UK) was used. The same fixation and staining protocol was used for immunocytochemistry and cytospins.

For immunocytochemistry, pericytes were seeded onto a 24-well plate (10,000 cells/well) with coverslips (diameter 13 mm; Van Waters & Rogers) placed at the well bottoms, and maintained in the culture until confluent.

For cytospin preparations, 2 × 10^5^ pericytes sorted from 17-week-old fetal kidneys were centrifuged and immediately suspended in PBS/2% FBS (5 × 10^4^ cells/100 μl). Subsequently, cells were loaded into cytospin cuvettes and centrifuged for 3 minutes at 300*g*.

Sections of paraffin-embedded adult human kidneys were dewaxed in xylene and rehydrated through a graded series of alcohol to water. For fluorescent imaging, overnight incubations at 4 °C were performed sequentially with antirenin (1:100), anti-CD146 (Abcam 1:50), and anti-CD34 antibody (BD Pharmingen 1:100). For better visualization of tissue morphology, antirenin (60 minutes at room temperature, 1:100) staining was performed with 3,3′-diaminobenzadine (Dako, Cambridge, UK).

Negative controls were performed for each immunohistochemistry experiment, consisting of isotype-matched negative control or omitting the primary antibody.

### Renin induction

Confluent pericyte cultures were serum-starved (DMEM/1% PS) for 24 hours. Renin induction was stimulated with cAMP inducers (10-μm forskolin; Cell Signalling, Leiden, Netherlands, and 100 μm IBMX; Sigma-Aldrich) in DMEM/1% PS. Controls consisted of vehicle (medium/ethanol/0.01% dimethyl sulfoxide) and serum-free medium with no additives (DMEM/1% PS). After 24 hours, culture media were collected for renin activity assay. After a PBS wash, cells were harvested for RNA extraction.

### Renin activity assay

Renin activity in media from primary pericytes cultures was determined by radioimmunoassay of angiotensin I generated by incubation with angiotensinogen as previously described. Media aliquots derived from renin-induced pericyte primary cultures and media containing 0 to 400 pmol angiotensin I standards (Sigma-Aldrich) were incubated at 37 °C and at 0 °C for 1 hour with an assay buffer (50-mM Tris/hydrogen chloride, 0.2% neomycin sulphate, and 0.1% human serum albumin, pH 7.4) that contained porcine renin substrate and angiotensin I antibody. Renin activity was calculated by subtracting the amount of angiotensin I in samples incubated at 0 °C from values of samples incubated at 37 °C.

### Electron microscopy

Control and induced-treated pericytes cultured on polyester filters were fixed with 3% paraformaldehyde/0.05% glutaraldehyde in 0.1-M phosphate buffer (pH 7.2) for 10 minutes at 37 °C. Filters were stained with uranyl acetate (2% weight/volume in distilled water), dehydrated through increasing concentrations of methanol (70%–100%) at −20 C, and embedded in LR Gold resin (London Resin Company, Reading UK). Ultrathin sections (50–80 nm) were prepared with a Reichart-Jung ultracut microtome. For immunogold labeling of renin and the pericyte marker NG2, sections were incubated for 2 hours at 37 °C with primary antihuman renin (1:1000) or NG2 antibodies (1:500), then for 1 hour with 15-nm Protein A gold-conjugate (1:60; British Biocell, Cardiff, Wales, UK). No labeling was observed in negative control sections in which the primary antibody was replaced with PBS/0.1% egg albumin. Sections were counterstained with lead citrate and uranyl acetate, and examined on a JOEL 1010 TEM (JOEL USA Inc., Peabody, Massachusetts, USA).

### Statistics

Data are expressed as mean ± SE. One-way analysis of variance was used to determine statistical significance.

## Disclosure

All the authors declared no competing interests.

## Supplementary Material

Table S1 List of primers used for real-time polymerase chain reaction Table S1
