# Supplementary material for: Human kidney pericytes produce renin
Source: Kidney Int. 2016 Dec;90(6):1251–61. doi: 10.1016/j.kint.2016.07.035 (PMC5126097; doi:10.1016/j.kint.2016.07.035)
Supplement: Table S1 — List of primers used for real-time polymerase chain reaction [file mmc1.docx]

**Supplementary Table S1. List of primers used for RT-PCR.**

| β -actin | Forward | CCTCGCCTTTGCGCATCC-3’ |
| --- | --- | --- |
|  | Reverse | GGAATCCTTCTGACCCATGC-3’ |
| CD146 | Forward | AAGGCAACCTCAGCCATGTCG |
|  | Reverse | CTCGACTCCACAGTCTGGGAC |
| NG2 | Forward | GCTTTGACCCTGACTATGTTGGC |
|  | Reverse | AAGCCAATGCGGTTGTTACGC |
| PDGFR -β | Forward | CAGTAAGGAGGACTTCCTGGAG |
|  | Reverse | CCTGAGAGATCTGTGGTTCCAG |
| CD34 | Forward | CATCACTGGCTATTTCCTGAT |
|  | Reverse | AGCCGAATGTGTAAAGGACAG |
| CD56 | Forward | GATTTTGCCTATCCCAGTGCC |
|  | Reverse | CATACTTCTTCACCCACTGCT |
| Foxd1 | Forward | CCGGCTCCTTTTCTCGTCTT |
|  | Reverse | ACGTCAAGGGAGCCTCTAGT |
| CRIM1 | Forward | ATGTGTGCCCTGATCACCTG |
|  | Reverse | CGCTGTTATTGCGGGACAAG |
